# FLT3 and IRAK4 Inhibitor Emavusertib in Combination with BH3-Mimetics in the Treatment of Acute Myeloid Leukemia

**DOI:** 10.3390/cimb46040184

**Published:** 2024-03-29

**Authors:** Katja Seipel, Harpreet Mandhair, Ulrike Bacher, Thomas Pabst

**Affiliations:** 1 Department for Biomedical Research, University of Bern, 3008 Bern, Switzerland; harpreet.mandhair@unibe.ch; 2 Department of Hematology, University Hospital Bern, 3010 Bern, Switzerland; veraulrike.bacher@insel.ch; 3 Department of Medical Oncology, University Hospital Bern, 3010 Bern, Switzerland

**Keywords:** acute myeloid leukemia (AML), B-cell lymphoma 2 (BCL2), BCL2 homology domain 3 (BH3), interleukin-1 receptor-associated kinase 4 (IRAK4), leukocyte integrin CD11B, cell surface glycoprotein CD34, stem cell factor receptor c-KIT (CD117), heat-shock protein 90 (HSP90), myeloid cell leukemia 1 (MCL1), IRAK4 inhibitor emavusetib (CA4948), HSP90 inhibitor PU-H71, MCL1 inhibitor S63845, BCL2 inhibitor venetoclax

## Abstract

Targeting the FLT3 receptor and the IL-1R associated kinase 4 as well as the anti-apoptotic proteins MCL1 and BCL2 may be a promising novel approach in the treatment of acute myeloid leukemia (AML). The FLT3 and IRAK4 inhibitor emavusertib (CA4948), the MCL1 inhibitor S63845, the BCL2 inhibitor venetoclax, and the HSP90 inhibitor PU-H71 were assessed as single agents and in combination for their ability to induce apoptosis and cell death in leukemic cells in vitro. AML cells represented all major morphologic and molecular subtypes, including *FLT3-ITD* and *NPM1* mutant AML cell lines and a variety of patient-derived AML cells. Emavusertib in combination with MCL1 inhibitor S63845 or BCL2 inhibitor venetoclax induced cell cycle arrest and apoptosis in MOLM-13 cells. In primary AML cells, the response to emavusertib was associated with the presence of the *FLT3* gene mutation with an allelic ratio >0.5 and the presence of *NPM1* gene mutations. S63845 was effective in all tested AML cell lines and primary AML samples. Blast cell percentage was positively associated with the response to CA4948, S63845, and venetoclax, with elevated susceptibility of primary AML with blast cell fraction >80%. Biomarkers of the response to venetoclax included the blast cell percentage and bone marrow infiltration rate, as well as the expression levels of CD11b, CD64, and CD117. Elevated susceptibility to CA4948 combination treatments with S63845 or PU-H71 was associated with *FLT3*-mutated AML and CD34 < 30%. The combination of CA4948 and BH3-mimetics may be effective in the treatment in *FLT3*-mutated AML with differential target specificity for MCL1 and BCL2 inhibitors. Moreover, the combination of CA4948 and PU-H71 may be a candidate combination treatment in *FLT3*-mutated AML.

## 1. Introduction

The fms-like tyrosine kinase receptor 3 (FLT3) gene is overexpressed in up to 93% and mutated in over 30% of primary acute myeloid leukemia (AML) [1]. FLT3 signaling pathways are highly active in AML cells, with consequential induction of protein translation and cell proliferation as well as reduced apoptosis. Several FLT3 kinase inhibitors have been approved for the treatment of *FLT3*-mutated AML; however, the therapeutic effects may be short-lived due to the emergence of adaptive resistance [2]. Therapy resistance occurs through target-dependent mechanisms resulting from point mutations in the FLT3 kinase domain or through target-independent mechanisms, such as an alternate activation of survival and proliferation pathways [3], including the elevated expression of anti-apoptotic proteins of the B-cell lymphoma 2 (BCL2) family. MCL1 and BCL2 proteins are both frequently overexpressed in AML and critical for the survival of AML cells and leukemic stem cells [4]. While the BCL2 inhibitor venetoclax, in combination with azacitidine, decitabine, or low-dose cytarabine (LDAC), has been approved for the treatment of adults with newly diagnosed AML [5], the MCL1 inhibitor S63845 has been evaluated as a candidate treatment in AML in combination with the MEK inhibitor trametinib, the BMI1 inhibitor PTC596, or the HSP90 inhibitor PU-H71 in preclinical studies [6,7,8].

Interleukin-1 receptor-associated kinases are emerging therapeutic targets in hematologic malignancies [9]. IRAK4 is a key mediator in interleukin- and Toll-like receptor (TLR) signaling in the innate immune response [10]. Recruitment and subsequent activation of IRAK4 upon interleukin 1 (IL-1) binding to IL-1R or TLR4 stimulation by bacterial lipopolysaccharides (LPS) can be mediated by the myeloid differentiation primary response 88 (MYD88) adaptor protein [11]. In AML cells, LPS binding to TLR4 promotes cell proliferation, inhibits apoptosis, and increases resistance to chemotherapy [12]. Like FLT3, TLRs and their associated signal transducers are frequently overexpressed and/or constitutively activated in hematological malignancies [13]. IRAK4 inhibitors were originally developed for the treatment of chronic inflammatory auto-immune diseases such as rheumatoid arthritis [14]. Multikinase FLT3-IRAK1/4 inhibitors have been developed to overcome the adaptive resistance of myeloid cells to specific FLT3 inhibitors [15]. Pacritinib, a multi-kinase inhibitor of IRAK1, JAK2, and FLT3, demonstrated anti-leukemic activity in combination with chemotherapy in patients with *FLT3* mutations [16,17]. Emavusertib (CA4948), a multi-kinase inhibitor of IRAK4 and FLT3, has been proposed for the treatment of B-cell lymphoma and myeloid malignancies [10,18]. Emavusertib is currently being evaluated in a phase 1/2 clinical trial in R/R AML as a monotherapy and in combination with azacitidine or venetoclax (NCT04278768) [19]. New insights into the biology of IRAK4, the development of IRAK4 inhibitors, and synergies with established treatments such as chemotherapy and targeted inhibitors were discussed at the first symposium on IRAK4 in cancer [20].

HSP90 inhibitors may destabilize oncoproteins associated with the cell cycle, angiogenesis, RAS-MAPK activity, histone modification, kinases, and growth factors [21,22]. However, due to toxicity and limited efficacy, no HSP90 inhibitor has been approved for clinical use as single agent [23]. Combining HSP90 inhibitors with other anticancer therapies might be a more advisable strategy to reduce toxicity and increase efficacy [24]. The combination of the HSP90 inhibitor PU-H71 and the MCL1 inhibitor S63845 may be a candidate treatment for *FLT3*-mutated AML with moderate CD34 positivity, while the combination of HSP90 inhibitor PU-H71 and BCL2 inhibitor venetoclax may be more effective in the treatment of primitive AML with high CD117 and low CD11b positivity [8].

In this study, we assessed the multikinase inhibitor emavusertib (CA4948), the MCL1 inhibitor S63845, the BCL2 inhibitor venetoclax (ABT-199), and the HSP90 inhibitor PU-H71 as single agents and in combination for their ability to induce apoptosis and cell death in leukemic cells in vitro. AML cells represented all major morphologic and molecular subtypes, including *FLT3-ITD* and *FLT3* wild type, *NPM1* mutant and wild type, as well as *TP53* mutant and wild type AML cell lines and primary AML cells.

## 2. Materials and Methods

### 2.1. Patient Samples

Mononuclear cells of AML patients diagnosed and treated at the University Hospital, Bern, Switzerland, between 2015 and 2022 were included in this study. Informed consent was obtained according to the Declaration of Helsinki, and the studies were approved by decisions of the Ethics Committee of the Canton of Bern, Switzerland. Peripheral blood mononuclear cells (PBMCs) and bone marrow mononuclear cells (BMMCs) were collected at the time of diagnosis before the initiation of treatment. AML cells were analyzed at the central hematology laboratory of the University Hospital Bern according to current practice and techniques [25], including mutational screening of *FLT3*, *NPM1*, and *TP53* genes and conventional karyotype analysis of at least 20 metaphases. All samples were analyzed by NGS sequencing of the myeloid panel genes, encoding spliceosome components (U2AF1, SF3B1, SRSF2, ZRSR2), epigenetic modifiers (TET2, DNMT3A, BCOR, ASXL1, IDH1, IDH2), cohesins (STAG2, RAD21, SMC3), transcription factors (TP53, RUNX1, WT1, ETV6), signaling molecules (FLT3, NF1, NRAS, CBL, PTPN11, JAK2), and chromatin modifiers (ASXL1, EZH2, SUZ12). Flow-cytometry immuno-phenotyping was performed according to the international consensus with CD markers in the initial evaluation of myeloid leukemia, including CD7, CD11b, CD13, CD14, CD15, CD16, CD33, CD34, CD45, CD56, CD117, and HLA-DR.

### 2.2. Cell Lines and Cell Culture

Human AML cell lines OCI-AML3 (AML-M4, *FLT3*wt, DNMT3A R882C, *NPM1*mut, *TP53*wt), MOLM-13 (AML-M5, t (9;11), *FLT3*-ITD, *TP53*wt), SKM-1 (AML-M5, *FLT3*wt, *TP53*mut), and ML-2 (AML-M4, t (6;11), *FLT3*wt, *TP53*wt) cells were supplied by the Leibniz Institute DSMZ, German Collection of Microorganisms and Cell Cultures. AML cells were grown in RPMI 1640 media (SIGMA-ALDRICH, St. Louis, MO, USA) supplemented with 20% fetal bovine serum (FBS, Biochrom GmbH, Berlin, Germany) in a standard cell culture incubator at 37 C with 5% CO_2_. The human bone marrow stroma cell line HS-5 was supplied by the American Type Culture Collection (ATCC) (ATCC^®^ CRL-11882™). HS-5 cells were grown in DMEM media (SIGMA-ALDRICH, St. Louis, MO, USA) supplemented with 10% fetal bovine serum (FBS, Biochrom GmbH, Berlin, Germany). The HS-5 cells secreted granulocyte colony-stimulating factor (G-CSF), granulocyte-macrophage-CSF (GM-CSF), macrophage-CSF (M-CSF), Kit ligand (KL), macrophage-inhibitory protein-1 alpha, interleukin-1 alpha (IL-1alpha), IL-1beta, IL-1RA, IL-6, IL-8, IL-11, and leukemia inhibitory factor (LIF). For the co-culture assays, HS-5 cells were plated on six-well plates on day 1. On day 2, Nunc 0.4 uM cell culture inserts (ThermoFisher, Roskilde, Denmark) were placed over the HS-5 feeder layer and AML cells were filled into the cell culture inserts. On day 3, AML cells were collected from the six-well inserts and replated on 96-well plates before the addition of compounds. Cytotoxicity assays were performed on day 4.

### 2.3. Cytotoxicity Assays

The IRAK4 inhibitor Emavusertib (CA4948), the MCL1 inhibitor S63845, the HSP90 inhibitor PU-H71, and lipopolysaccharides (LPS) were purchased from MedChem-Express (Monmouth Junction, NJ, USA). A stock solution of Venetoclax (ABT-199) was prepared by dissolving a tablet in DMSO (Venclexta^®^, Abbvie Inc., North Chicago, IL, USA). Cryopreserved patient-derived mononuclear cells were thawed and cultured overnight prior to the start of treatment. AML cells were plated at a density of 5 × 10^5^/mL. Cell viability was determined after 20 h of treatment using the MTT-based cell proliferation kit I (Roche Diagnostics GmbH, Mannheim, Germany). This time point was selected because the cellular responses were effectual for the calculation of combination indexes after 20 h of treatment with two compounds in leukemic cells. For AML cell lines, four independent assays (biological replicates) with four measurements (technical replicates) per dosage were performed. For hematological patient samples, two independent assays with three technical replicates per dosage were performed. For the calculation of combination indexes, three dosages of CA4948 and two dosages of the other compounds were applied alone and in combination. Combination indexes were calculated on Compusyn software version 1.0 (Informer technologies, Inc., multinational (Los Angeles, CA, USA)) according to Chou Talalay [26]. Data are depicted as XY graphs, column plots, or scatter plots with mean values and SD. Statistical analysis was carried out using GraphPad Prism version 10 (GraphPad, San Diego, CA, USA) in grouped analysis, and the significance was calculated using the *t*-test for column graphs or the Mann–Whitney test for scatter plots.

### 2.4. Imaging Cytometry

Imaging cytometry was carried out on an NC-3000 cell analyzer (ChemoMetec, Allerod, Denmark), with reagents supplied by ChemoMetec. To determine the induction of apoptosis, cells were stained with AnnexinV-CF488A conjugate (Biotium, Fremont, CA, USA) in AnnexinV buffer and Hoechst 33,342 (10 μg/mL) for 15 min at 37 °C, followed by several washes. Propidium iodide was added shortly before imaging. According to Annexin V and PI staining intensity, cells were classified as vital (Ann lo, PI lo), early apoptotic (Ann hi, PI lo), late apoptotic (Ann hi, PI hi) or necrotic (Ann lo, PI hi). For cell cycle analysis, cells were incubated in lysis buffer with DAPI (10 μg/mL) for 5 min at 37 C before imaging on the NC-3000 cell analyzer. According to DAPI staining intensity, cells were classified as subG1 (<2 N), G0/G1 (2 N), S phase (2–4 N), or G2 phase (4 N). Statistical analysis was performed using the unpaired *t*-test on GraphPad Prism version 10. Data are depicted as stacked column bar graphs.

## 3. Results

### 3.1. Inverse Response of AML Cell Lines to Emavusertib (CA4948) Monotherapy

To determine the susceptibility to emavusertib (CA4948), a variety of AML cell lines were subjected to in vitro cytotoxicity assays. AML cells represented the major morphologic and molecular subtypes, including *FLT3-ITD* and *FLT3* wild type, *NPM1* mutant and wild type, as well as *TP53* mutant and wild type AML cell lines. AML cells were treated for 20 h in dose escalation experiments before the cell viability assessment.

**Figure 1:**
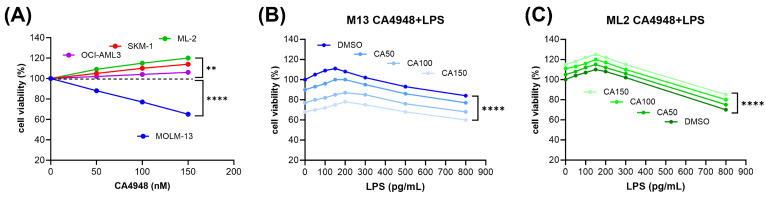
In vitro dose response to emavusertib (CA4948) treatment in AML cell lines. AML cells were treated with the dual IRAK4 and FLT3 inhibitor CA4948 at the indicated dosages for 20 h (**A**). Susceptibility of AML cells to CA4948 under exposure to lipopolysaccharides (**B**,**C**). Cell viability was determined in AML cells after exposure to 50–250 pg/mL LPS and 50 to 150 nM CA4948 in FLT3-ITD positive MOLM-13 (**B**) and FLT3 wild-type ML-2 (**C**) cells. Cell viability data are average values of multiple repeat measurements per dosage. The standard deviation was 3–6%. Significance of differences denoted for *p* < 0.01 (**); and *p* < 0.0001 (****).

A dose-dependent decrease in cell viability in the presence of CA4948 was detected in the *FLT3-ITD*-positive MOLM-13 cells, with IC50 at 150 nM. In contrast, there was a dose-dependent increase in cell viability in the presence of CA4948 in the *FLT3* wild-type cell lines ML-2, OCI-AML3, and SKM-1 (Figure 1A).

### 3.2. Susceptibility of AML Cells to Emavusertib (CA4948) Treatment under Exposure to Lipopolysaccharide

**Figure 2:**
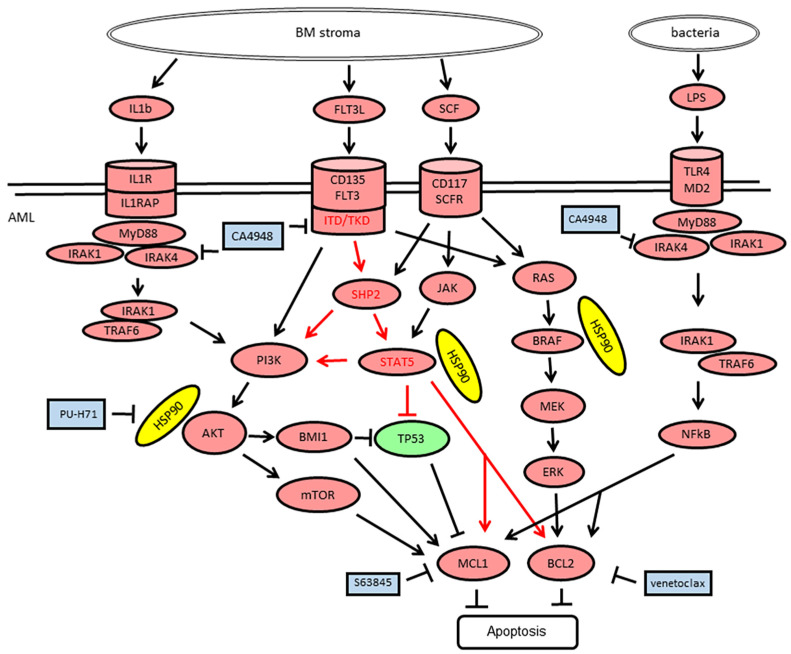
Schematic presentation of the FLT3 and SCFR (CD117, c-KIT) as well as IL1R and TLR4 signaling pathways and downstream effects. FLT3-ITD and FLT3-TKD are constitutively active growth factor receptors. SCFR (c-KIT) is an inducible growth factor receptor activated by stem cell factor (SCF). Both tyrosine receptor kinases activate PI3K-AKT, RAS-MEK-ERK, and STAT5, leading to cell growth and proliferation via inhibition of the tumor suppressor TP53 and induction of the apoptosis regulators MCL1 and BCL2. IL1R and TLR4 are inducible receptors signaling via MyD88 and IRAK4. HSP90 protein can bind and stabilize client proteins, including AKT, BCL2, FLT3, JAK, MDM2, STAT5, SHP2, and BRAF. Hsp90 proteins are indicated in yellow, oncogenic protein functions in red, tumor suppressor functions in green ovals, and targeted inhibitors in blue rectangles. Sharp arrows and blunt arrows indicate target induction and inhibition, respectively.

Lipopolysaccharide (LPS) is an endotoxin derived from the outer membrane of Gram-negative bacteria, a pathogen-associated molecular pattern (PAMP) recognized by Toll-like receptors (TLRs) and other pattern recognition receptors (PRRs) [27]. In monocytes, LPS binding promotes the secretion of pro-inflammatory cytokines, including IL-1β and IL-6 [28]. In AML cells, LPS binding to TLR4 promotes cell proliferation, inhibits apoptosis, and increases resistance to chemotherapy [12]. Plasma LPS concentrations have been reported to be in the range of 10 to 50 pg/mL in healthy humans, 50 to 250 pg/mL in the peripheral blood of AML patients [23], and 500 pg/mL in patients with sepsis [24]. AML cell lines were exposed to LPS within the physiological concentration ranges and subjected to in vitro cytotoxicity assays. Exposure to LPS in the range of 50 to 250 pg/mL induced cell viability of AML cells in a dose-dependent manner in both *FLT3-ITD*-positive MOLM-13 (Figure 1B) and *FLT3* wild-type ML-2 cells (Figure 1C). Exposure to LPS over 500 pg/mL reduced the cell viability of AML cells in a dose-dependent manner. CA4948 treatment of AML cells exposed to LPS led to reduced cell viability in MOLM-13 cells (Figure 1B) and enhanced cell viability in ML-2 cells (Figure 1C). The combined effects appeared to be additive, not synergistic, in nature, indicating an absence of cooperative interactions on the molecular level. LPS is one of several ligands binding to the TLR4/MD2 receptor complex (Figure 2).

### 3.3. Emavusertib Combination Treatments in AML Cell Lines

**Figure 3:**
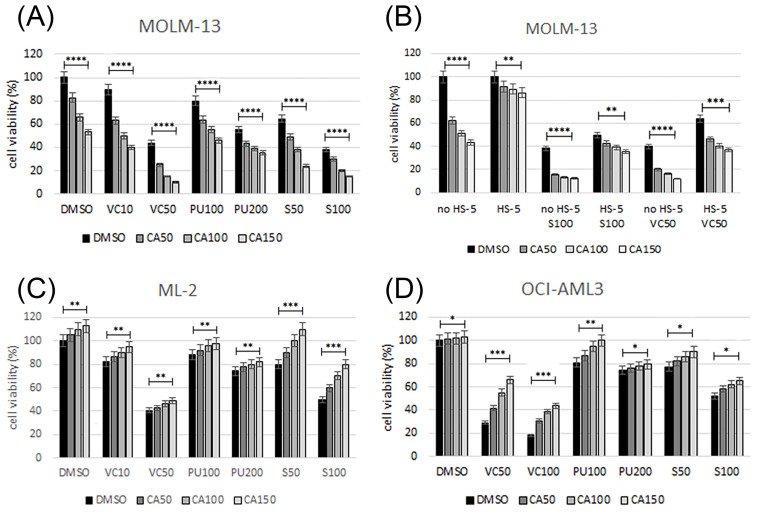
Susceptibility of AML cell lines to various treatment combinations. Cell viability was determined in AML cell lines MOLM-13, ML-2, and OCI-AML3 after 20 h of treatment with single compounds and in combination with 50–150 nM CA4948 (CA) and 10–100 nM venetoclax (VC100), 100–200 nM PU-H71 (PU), or 50–100 nM S63845 (S100). Susceptibility of MOLM-13 cells to various treatment combinations in the absence or presence of HS-5 bone marrow stroma (**B**). Cell viability data are average values of multiple repeat measurements per dosage. Synergistic effects of combination treatments are depicted in MOLM-13 (**A**). Oppositional effects in ML-2 (**C**) and OCI-AML3 (**D**) cells. Cell viability data are average values of multiple repeat measurements per dosage. Significance of differences denoted for *p* < 0.05 (*); *p* < 0.01 (**); *p* < 0.001 (***); and *p* < 0.0001 (****).

In order to define effective treatment combinations, we focused on inhibitors expected to elicit synergistic effects in combination with the IRAK4/FLT3 inhibitor CA4948 based on previous studies with BCL2, MCL1, and HSP90 inhibitors (Figure 2). Cell viability was determined in AML cell lines treated with increasing dosages of single compounds, as well as in combination treatments using the IRAK4 inhibitor CA4948 and a variety of targeted therapies, including the BCL2 inhibitor venetoclax, the MCL1 inhibitor S63845, and the HSP90 inhibitor PU-H71. *FLT3-ITD*-positive MOLM-13 cells were susceptible to CA4948, S63845, venetoclax, and PU-H71, with enhanced effects on cell viability in the combination treatments (Figure 3A). Combination index (CI) values were calculated according to Chou-Talalay [26]. In MOLM-13 cells, the combination indexes indicated synergistic effects (CI 0.5–0.7) for the combination CA4948 and S63845, or venetoclax and additive effects (CI 0.9–1.1) for the combination CA4948 and PU-H71. Cell viability was determined in MOLM-13 cells grown in the absence or presence of bone marrow stroma cells secreting granulocyte and macrophage colony-stimulating factors, as well as other cytokines, including IL-1 and stem cell factor SCF (Figure 2). In the presence of bone marrow stroma, MOLM-13 cells were protected from the cytotoxic effects of CA4948, S63845, and venetoclax, and combination treatments were less effective (Figure 3B). *FLT3* wild-type OCI-AML3 and ML-2 cells were susceptible to S63845, venetoclax, and PU-H71, but, with a dose-dependent increase in cell viability in the presence of CA4948 in the combination treatments (Figure 3C,D).

### 3.4. Emavusertib Treatment Induced Cell Cycle Arrest, Apoptosis and Cell Death

**Figure 4:**
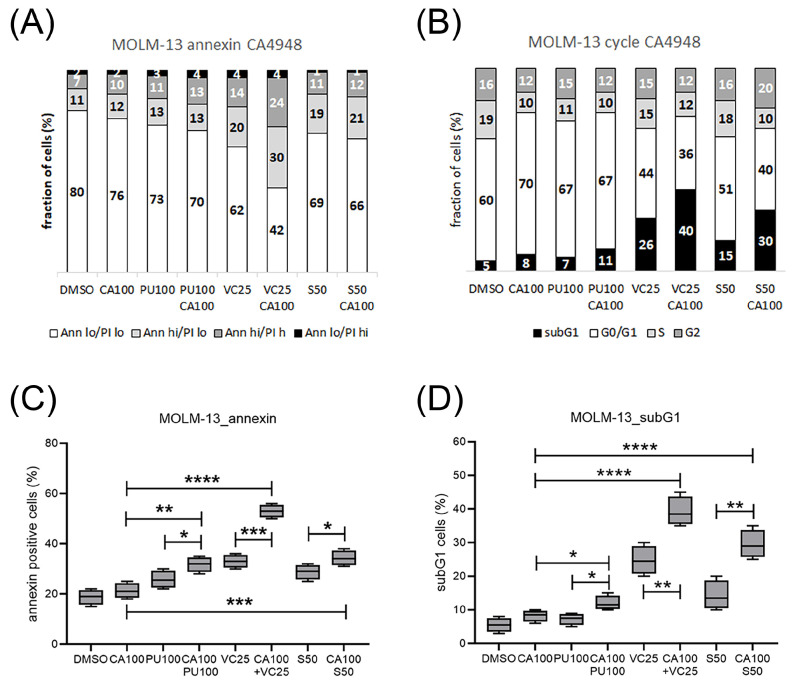
Induction of cell cycle arrest, apoptosis, and cell death in AML cells treated with CA4948 in combination with PU-H71, S63845, or venetoclax. Cytometric analysis of MOLM-13 cells treated with 100 nM PU-H71 (PU), 50 nM S63845 (S), or 25 nM venetoclax (VC) and stained with Annexin-V and PI (**A**,**C**) or DAPI (**B**,**D**). According to Annexin V and PI staining intensity, cells were classified as vital (Ann lo, PI lo), early apoptotic (Ann hi, PI lo), late apoptotic (Ann hi, PI hi), or necrotic (Ann lo, PI hi). According to DAPI staining intensity, cells were classified as subG1 (<2 N), G0/G1 (2 N), S phase (2–4 N), or G2 phase (4 N). Significance of differences denoted for *p* < 0.05 (*); *p* < 0.01 (**); *p* < 0.001 (***); and *p* < 0.0001 (****).

The effects of treatment with CA4948 and S63845, venetoclax, or PU-H71, alone and in combination, on the induction of apoptosis, cell cycle arrest, and cell death were determined in MOLM-13 cells by cytometric analysis. Apoptosis was induced in MOLM-13 cells treated with CA4948, PU-H71, S63845, or venetoclax, with enhanced effects in the combination treatments (Figure 4A,C). G1 cell cycle arrest was induced in CA4948- and PU-H71-treated MOLM-13 cells (Figure 4B). Cell death was induced in MOLM-13 cells treated with CA4948, PU-H71, S63845, or venetoclax, with enhanced effects in the combination treatments (Figure 4B,D).

### 3.5. Emavusertib Combination Treatments in Primary AML Cells In Vitro

**Figure 5:**
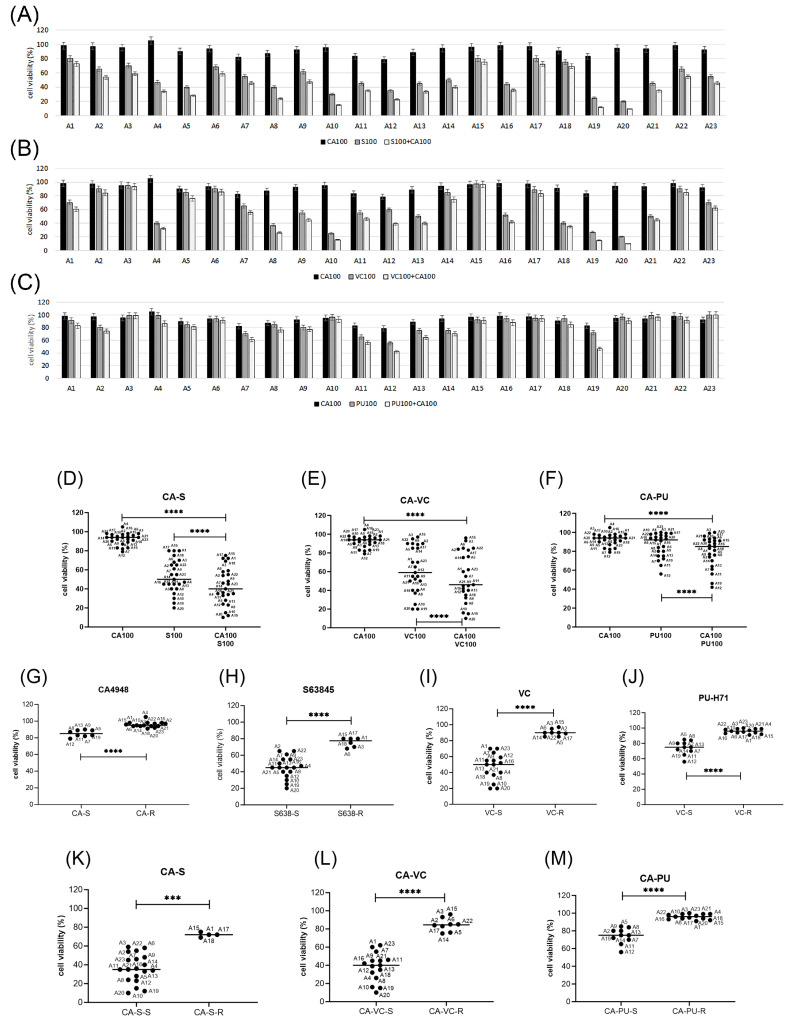
Primary AML cells’ in vitro response to Emavusertib and combination treatments. Cell viability was determined in mononuclear cells isolated from the peripheral blood or bone marrow of 23 AML patients (A1 to A23) after 20 h of treatment. (**A**) Primary AML cells treated with 100 nM CA4948 (CA100) and 100 nM S63845 (S100), alone and in combination. (**B**) Primary AML cells treated with 100 nM CA4948 (CA100) and 100 nM venetoclax (VC100), alone and in combination. (**C**) Primary AML cells treated with 100 nM CA4948 (CA100) and 100 nM PU-H71 (PU100), alone and in combination. Treatment for 20 h with 100 nM CA4948 and 100 nM S63845 (**D**), 100 nM CA4948 and 100 nM venetoclax (**E**), and 100 nM CA4948 and 100 nM PU-H71 (**F**). Significance was calculated by paired *t*-test. The patient samples were sorted into two groups, susceptible (S) and resistant (R), in a single compound treatment with CA4948 (**G**), S63845 (**H**), venetoclax (**I**), and PU-H71 (**J**), as well as in different combination treatments with CA4948 and S63845 (**K**), CA4948 and venetoclax (**L**), and CA4948 and PU-H71 (**M**). Significance of differences in median values was calculated using the Mann–Whitney test. Significance is denoted for *p* < 0.001 (***) and *p* < 0.0001 (****).

After initial studies in AML cell lines, the treatment combinations of emavusertib (CA4948) with S63845, venetoclax, or PU-H71 were applied to patient-derived mononuclear cells isolated from peripheral blood (PB) or bone marrow (BM). A total of twenty-three primary AML cells were subjected to single compound and combination treatments, with eleven *FLT3* mutated and twelve *FLT3* wild-type primary AML. In vitro cytotoxic effects were mild to moderate in CA4948 and PU-H71 monotherapy and moderate to strong in S63845 or venetoclax monotherapy, with enhanced effects in the combination treatments (Figure 5A–C).

**Figure 6:**
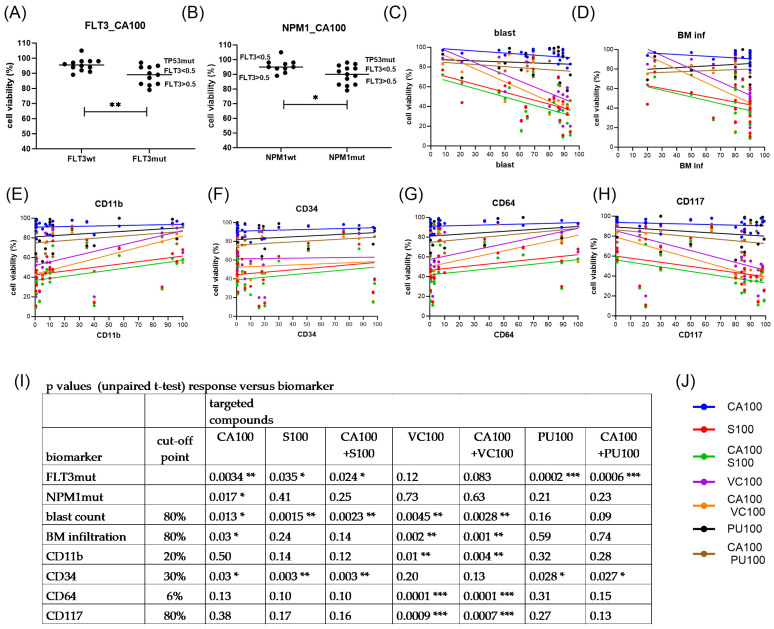
Biomarkers of response to emavusertib and combination treatments. Cell viability was determined in mononuclear cells isolated from AML patients’ peripheral blood or bone marrow after 20 h of treatment with 100 nM CA4948 (CA100), 100 nM S63845 (S100), 100 nM venetoclax (VC100), and 100 nM PU-H71 (PU100), alone and in combination. Response was correlated to *FLT3* gene mutation status (**A**), *NPM1* gene mutation status (**B**), blast cell percentage (**C**), bone marrow infiltration (**D**), CD11b (**E**), CD34 (**F**), CD64 (**G**), and CD117 (**H**). Best fit lines are according to simple linear regression analysis. Association of response and biomarkers was analyzed by unpaired *t*-test (**I**). Significance denoted for *p* < 0.05 (*); *p* < 0.01 (**); *p* < 0.001 (***). Legend for treatment combinations (**J**).

CA4948 monotherapy was effective in 35% of primary AML (Figure 5D). S63845 monotherapy was effective in all tested AML samples, with enhanced toxicity in 75% of primary AML (Figure 5E). Venetoclax monotherapy was effective in 65% of primary AML (Figure 5F), and PU-H71 monotherapy in 44% of primary AML (Figure 5G). The patient samples were sorted into response groups, susceptible (S) and resistant (R), treated with 100 nM CA4948 alone (Figure 6D) and in combination with 100 nM S63845 (Figure 5E), 100 nM venetoclax (Figure 6F), or 100 nM PU-H71 (Figure 6G). The cut-off values of cell viability were set arbitrarily, in CA4948 and PU-H71 monotherapy at 90%, venetoclax at 80%, and S63845 at 70%. In combination treatments, they were set to 85% (CA-PU), 70% (CA-VC), or 60% (CA-S). CA4948 and S63845 combination treatments were effective in all tested AML samples, with enhanced toxicity in 83% of primary AML (Figure 5H). CA4948 and venetoclax combination treatments were effective in 65% of primary AML (Figure 5I), and CA4948 and PU-H71 in 44% of primary AML (Figure 5J).

### 3.6. Biomarkers of Response to Emavusertib and Combination Treatments in Primary AML Cells

Potential response markers were deduced from the correlation analysis of cell viabilities grouped according to diagnostic parameters, including gene mutation status; peripheral blood and bone marrow blast cell percentage; and expression levels of CD markers, including CD11b, CD34, CD64, and CD117 (c-KIT). The clinical characteristics of the hematological samples are listed in Table 1. The presence of a *FLT3* gene mutation was the major indicator of a response to CA4948, S63845, and PU-H71 treatment, with elevated susceptibility of AML cells with the *FLT3* gene mutation at an allelic ratio >0.5 (Figure 6A).

The presence of a *NPM1* gene mutation was positively associated with the response to CA4948 (Figure 6B). *NPM1* gene mutations were present in most of the *FLT3*-mutated AML (8/11) and a few of the *FLT3* wild-type primary AML (3/12). The one *FLT3* wild-type and *NPM1*-mutated sample with substantial response to CA4948 and S63845 (AML5) also carried a specific *PTPN11* gene mutation (E69K). The blast cell percentage was positively associated with response to CA4948, S63845, and venetoclax, along with elevated susceptibility of primary AML with a blast cell count >80% (Figure 6C). Bone marrow (BM) infiltration was positively associated with the venetoclax response, with elevated susceptibility of primary AML with BM infiltration of >80% (Figure 6D). CD11b was negatively associated with the venetoclax response, with elevated susceptibility of primary AML with CD11b < 20% (Figure 6E). CD34 was negatively associated with the response to CA4948, S63845, and PU-H71, with elevated susceptibility of primary AML samples with CD34 < 30% (Figure 6F). CD64 was negatively associated with the venetoclax response, with elevated susceptibility of primary AML samples with CD64 < 10% (Figure 6G). CD117 was positively associated with the venetoclax response, with elevated susceptibility of primary AML samples with CD117 > 80% (Figure 6H). Biomarkers of the responses to individual treatments and statistical significances are listed in Figure 6I. Two primary AMLs carried *TP53* gene mutations, one in a *FLT3* wild-type AML (A1) and the other in a *FLT3-TKD* AML (A2). Both *TP53*-mutant AMLs were resistant to CA4948 monotherapy, but susceptible to S63845 and venetoclax, with enhanced effects in the combination treatments. Mutations in spliceosome genes *U2AF1* and *SF3B1* have been linked to the expression of oncogenic IRAK4 isoforms in myeloid malignancies [29,30]. *U2AF1* gene mutations were present in one *FLT3* wild-type AML (A3) and in one *FLT3-ITD* AML (A7), while a *SF3B1* gene mutation was present in one *FLT3* wild-type AML (A22). Of the three primary AMLs with *U2AF1* mutations, only the *FLT3-ITD*-positive AML was susceptible to CA4948. Mutations in the spliceosome gene *SRSF2* were present in five primary AMLs (A2, A10, A15, A18, A21), none of them susceptible to CA4948. The majority of primary AML (17/23) carried mutations in epigenetic modifiers (*TET2, DNMT3A, BCOR, ASXL1, IDH1, IDH2*). The majority of primary AMLs with wild-type status of epigenetic modifiers and *TP53* (4/5), and a minority of AMLs with mutations in epigenetic modifiers (4/17), were susceptible to CA4948.

In summary, emavusertib (CA4948) monotherapy appeared to be most effective in primary AML with *FLT3* and *NPM1* gene mutations and the absence of gene mutations in *TP53* and epigenetic modifiers, as well as moderate CD34 positivity. Combination treatments with CA4948 and venetoclax were effective in primary AMLs with elevated blast counts and bone marrow infiltration, as well as high CD117 expression and low levels of CD11b and CD64. Combination treatments with CA4948 and PU-H71 or S63845 were effective in *FLT3*-mutated AML with moderate CD34 positivity (<30%). A possible scenario of intracellular signaling in AML cells affected by targeted inhibition of IRAK4, FLT3, HSP90, BCL2, and MCL1 is presented in Figure 2.

## 4. Discussion

In order to determine the susceptibility of AML cell lines to the IRAK4 inhibitor emavusertib (CA4948), an initial dose escalation screening was performed in AML cell lines. Cytotoxic effects were present in the *FLT3-ITD*-positive cell line MOLM-13, with a dose-dependent decrease in cell viability. In contrast, there was a complete absence of cytotoxic effects in three *FLT3* wild-type cell lines, OCI-AML3, ML-2, and SKM-1, treated with 50–250 nM CA4948 with a dose-dependent increase in cell viability. A possible molecular mechanism underlying the accelerated cell growth may be CA4948-induced IRAK1 activation in *FLT3* wild-type AML cells, as IRAK4 inhibition can lead to functional compensation of IRAK1 in THP1 cells [31] indicating a requirement for cotargeting IRAK1 and IRAK4 in the treatment of *FLT3* wild-type AML.

In AML cells, LPS binding to TLR4 may promote cell proliferation, inhibit apoptosis, and increase resistance to chemotherapy [10]. Indeed, cell viability was induced in MOLM-13 and ML-2 AML cells exposed to bacterial LPS, a TLR4 agonist. Plasma LPS concentrations have been reported to be in the range of 10 to 50 pg/mL in healthy humans, 50 to 250 pg/mL in the peripheral blood of AML patients [23], and 500 pg/mL in patients with sepsis [24]. In AML patients, the intestinal barrier function may be compromised, leading to an increased translocation of bacteria and endotoxins into the blood. LPS may exacerbate leukemia progression in vitro and in vivo [32]. While cell viability was induced in AML cells exposed to intermediate LPS concentrations (50–250 pg/mL), cytotoxic effects were elicited in AML cells under LPS exposure over 500 pg/mL, equivalent to physiological conditions of bacterial sepsis. Sepsis has profound impacts on myeloid cell response. In the bone marrow, a progressive shift toward the release of immature myeloid cells (including myeloid-derived suppressor cells), at the expense of mature neutrophils, takes place [33,34]. Emavusertib (CA4948) treatment of AML cells exposed to LPS lead to reduced cell viability in *FLT3-ITD*-positive MOLM-13 cells and enhanced cell viability in *FLT3* wild-type ML-2 cells, indicating that a CA4948-induced compensatory IRAK1 activation occurs in *FLT3* wild-type cells.

Emavusertib as a monotherapy and in combination with azacitidine or venetoclax is currently being evaluated in a phase 1/2 clinical trial in R/R AML (NCT04278768) [19]. Combining emavusertib with other anticancer therapies may be a more effective treatment strategy. In order to define effective treatment combinations, we focused on inhibitors expected to elicit synergistic cytotoxic effects in combination with CA4948 based on previous studies with BCL2, MCL1, and HSP90 inhibitors [7,8,35]. *FLT3*-ITD-positive MOLM-13 cells were susceptible to CA4948, S63845, venetoclax, or PU-H71, with enhanced effects on cell viability in the combination treatments. After initial studies in AML cell lines, the treatment combinations of CA4948 with S63845, venetoclax, or PU-H71 were applied to primary AML cells. CA4948 and S63845 combination treatments were effective in all tested AML samples, with enhanced toxicity in 83% of primary AML; CA4948 and venetoclax combination treatments in 65% of primary AML; and CA4948 and PU-H71 combination treatments in 44% of primary AML. The in vitro concentrations of the tested inhibitors were in the range of physiologically relevant concentrations for all compounds. Emavusertib plasma concentrations of 1–5 μg/mL (2–10 μM) were detected in patients receiving 50 to 400 mg BID [18]. Venetoclax plasma concentrations of 1–3 μg/mL (1.2–3.5 μM) were observed in patients receiving 400 mg/day [36]. PU-H71 plasma concentrations of 1–5 μM were measured in patients infused with 100–400 mg/m^2^ [37]. S63845 displayed impressive potency at low nanomolar concentrations in preclinical in vitro and in vivo models of hematological malignancies, including MM, AML, CML, and c-MYC-driven Burkitt lymphoma [38].

Potential response markers were deduced from the correlation analysis of cell viabilities and grouped according to diagnostic parameters, including gene mutation status; peripheral blood and bone marrow blast cell percentage; and presence of CD markers, including CD11b, CD34, CD64, and CD117 (c-KIT). The *FLT3* gene mutation was the major biomarker of response to CA4948, S63845, and PU-H71 treatment, with elevated susceptibility of AML cells with the *FLT3* gene mutation at an allelic ratio of >0.5. Both *FLT3* and *NPM1* genes were mutated in the susceptible primary AML samples. The *FLT3* gene was overexpressed in up to 93% and mutated in over 30% of primary AML [1]. FLT3-ITD and FLT3-TKD are constitutively active growth factor receptors linked to the upregulation of BCL2 and MCL-1 expression in myeloid cells. BH3-mimetics are small compounds that antagonize anti-apoptotic BCL2 family proteins, and are specifically effective in apoptosis induction in *FLT3*-mutated AML cells [39]. *NPM1* gene mutations represent the most common genetic lesion in AML and cause aberrant localization of the mutant NPM1c protein [40]. NPM1c is exported to the cytoplasm, where it inhibits the tumor suppressor protein p53 by cytoplasmic retention. In a previous study, we found that the presence of the *NPM1* gene mutation was negatively associated with the susceptibility of FLT3-ITD AML cells to the FLT3 inhibitor midostaurin and the MDM2 inhibitor NVP-HDM201 [41]. In this preclinical study, however, the presence of the *NPM1* gene mutation was positively associated with the susceptibility of FLT3-ITD AML cells to the IRAK4 inhibitor CA4948. The presence of *NPM1* gene mutations has previously been correlated with the general response to TLR stimulation in AML cells [42]. Moreover, TLR4 may be a receptor for extracellular NPM1 protein [43]. Extracellular NPM1 protein acts as a potent inflammatory stimulator by promoting cytokine production (e.g., tumor necrosis factor-α (TNF-α)), which suggests that NPM1 acts as a damage-associated molecular pattern (DAMP). The wild-type NPM1 and mutant NPM1c proteins may differ in their abilities to activate TLR4 receptor signaling.

The surface marker CD34 was negatively associated with the response to CA4948, S63845, and PU-H71 treatment, with elevated susceptibility of primary AML samples with CD34 <30%. CD34 is a hematopoietic stem cell marker and may mediate the attachment to the bone marrow extracellular matrix and stromal cells [44]. Leukemic stem cells are a subpopulation of leukemia cells characterized by the CD34 + CD38- phenotype, and are considered to be resistant to standard treatment [45]. To address the adverse risk of CD34+ AMLs, the combination of the BMI1 inhibitor PTC596 with the MCL1 inhibitor S63845 may be a more effective treatment option [7]. We observed that three markers were associated with the venetoclax response: CD11b, CD64, and CD117. The markers CD11b and CD64 were negatively associated with the venetoclax response. Both CD11b (Integrin alpha M) and CD64 (FcγRI) are monocytic differentiation markers. CD11b+ CD64+ cells may exhibit resistance to venetoclax-based therapies due to a loss of BCL2 expression [46]. CD117 was positively associated with the venetoclax response. CD-117 is a tyrosine kinase receptor expressed on the majority of AML cells, where it induces signaling pathways similar to FLT3-ITD that are linked to the upregulation of BCL2 expression.

Mutations in the spliceosome genes *U2AF1* and *SF3B1* have been linked to the expression of oncogenic IRAK4 isoforms in myeloid malignancies [18,29,30], and may impose sensitivity to IRAK4 inhibition [31]. Mutations in epigenetic modifiers may also be linked to treatment responses to targeted therapies, e.g., enhanced sensitivity to venetoclax and azacitidine [47,48], venetoclax and bimiralisib [35], or AC-4-130 and S63845 [49]. In our preclinical study, the majority of primary AMLs with wild-type status of epigenetic modifiers and a minority of AMLs with mutations in epigenetic modifiers were susceptible to CA4948. In order to investigate a potential association of the emavusertib treatment response with spliceosome or epigenetic modifiers, preclinical studies in larger cohorts are required.

## 5. Conclusions

In this preclinical study, we assessed the IRAK4 inhibitor emavusertib (CA4948) in combination with the MCL1 inhibitor S63845, the BCL2 inhibitor venetoclax, and the HSP90 inhibitor PU-H71 in the treatment of acute myeloid leukemia and investigated the associated biomarkers of response. Our data suggest that treatment combinations with CA4948 and BH3-mimetics can effectively induce cell cycle arrest, apoptosis, and cell death in *FLT3*-mutated AML cells. In primary AMLs, the response to emavusertib was associated with the presence of the *FLT3* and *NPM1* gene mutations, blast cell percentage, and CD34 positivity. Biomarkers of the response to CA4948 combination treatment with S63845 included the blast cell percentage and CD34. Biomarkers of the response to CA4948 combination treatment with venetoclax included *FLT3* gene mutations as well as expression levels of CD11b, CD64, and CD117. The combination of emavusertib and BH3-mimetics may be effective in the treatment of AML with differential target specificities for MCL1 and BCL2 inhibitors. The combination of emavusertib and venetoclax may be an effective treatment in *FLT3*-mutated AML, where CD11b < 20%, CD64 < 10%, and CD117 > 80%. The combination of emavusertib and S63845 may be more effective in *FLT3*-mutated AMLs with blast cell counts >80% and CD34 < 30%.

## Figures and Tables

**Table 1:** Clinical characteristics of primary AML cells.

ID	Disease	Mutation Profile (VAF)	Karyotype	Source	CD34 %	CD117 %	Blast (%)	BM Inf (%)	CD11b %
A1	AML-M0	*TP53* G245S (92%)	complex	BM	97	93	74	80	5
A2	AML-M4	*FLT3*-TKD (0.565), *NPM1* (19%), *SRSF2* (47%), *TET2* Q1357fs (42%), *TET2* L1816fs (35%), *TP53* G245D (5%)	triple 8	PB	1	1	46	50	96
A3	AML-M5	*ASXL1* G646fs (41%), *KRAS* Q61R (39%), *SH2B3* L14P (17%), *TET2* Y1294H (47%), *U2AF1* Q157R (41%)	mono 7	BM	1	86	53	80	<1
A4	AML-M1/2	*CEBPA* K313del (49%), GATA2 A318G (14%), *TET2* H436fs (47%)	normal	BM	6	86	80	90	1
A5	AML-M4	*NPM1* (42%), *PTPN11* E69K (40%)	na	BM	6	16	65	65	86
A6	AML-M4	*ASXL1* G646fs (42%), *KRAS* G12D (64%), *TET2* R1214W (32%)	normal	PB	1	1	na	80	100
A7	AML-M1	*FLT3*-ITD (0,783), *BCOR* G1382fs (79%), *TET2* L1420* (39%), *U2AF1* S34F (40%)	triple 8	BM	18	30	na	na	12
A8	AML-M5	*FLT3*-ITD (0.5), *DNMT3A* R882H (48%), *NPM1* (49%), *IDH2* R140Q (49%)	normal	BM	1	86	87	90	<1
A9	AML-M1/2	*FLT3*-ITD (1.1), *NPM1* (43%)	normal	BM	4	86	50	90	8
A10	AML-NOS	*FLT3*-ITD (0.45), *TET2* R1261H (47%), *TET2* H1904R (48%), *SRSF2* (54%)	normal	PB	97	99	61	80	3
A11	AML-M1	*FLT3*-ITD (1.2), *NPM1* (35%), *WT1* R462P (47%)	normal	PB	19	90	86	90	10
A12	AML-M1/2	*FLT3*-ITD (1.0), *DNMT3A* R882C (50%), *NPM1* (39%), *RUNX1* P263S (51%)	normal	PB	10	84	92	90	1
A13	AML_M1	*FLT3*-ITD (0.56)	na	PB	<1	80	92	na	5
A14	AML-M4	*FLT3*-ITD (0.86), *DNMT3A* (45%), *NPM1* (36%), *SUZ12* (51%)	normal	PB	<1	84	21	20	7
A15	MDS-AML	*TET2* Q278* (42%), *TET2* M1701fs (35%), *NPM1* (31%), *ASXL1* (38%), *SRSF2* (42%)	triple 8	PB	51	50	69	80	35
A16	AML-M1	*NRAS* (45%), *DNMT3A* (46%), *NPM1* (22%), *RAD21* (44%)	+ 21	PB	<1	97	89	90	<1
A17	AML-M4	*NRAS* (32%), *PTPN11* F285I (46%), *DNMT3A* S243fs (45%), *DNMT3A* M880V (48%)	der (7;14)	PB	87	20	8	25	12
A18	AML-M0	*ASXL1* (48 %), *IDH2* (45 %), *RUNX1* (43%), *SRSF2* (34 %), *STAG2* (9 %)	+13	PB	98	94	64	80	1
A19	AML-M1	*FLT3*-ITD (1.0), *IDH2* (47%), *NPM1* (48%)	normal	PB	20	95	94	90	40
A20	AML-M1	normal	normal	PB	16	20	89	90	<1
A21	PV-AML	*FLT3*-TKD (0.16), *PTPN11* Y62D (18%), *IDH1* (42%), *NPM1* (38%), *SRSF2* (40%)	t(8;21), -Y	PB	78	37	83	90	32
A22	AML-M2	*SF3B1* (50%), *TET2* S689fs*4 (50%), *CBL* (87%)	normal	BM	76	64	69	80	25
A23	AML-M4	*DNMT3A* V895M (46%), *NPM1* (33%), *IDH2* R140Q (46%)	normal	PB	<1	30	86	85	57

Abbreviations: Acute myeloid leukemia (AML), bone marrow (BM), bone marrow infiltration (BM Inf), peripheral blood (PB), polycytemia vera (PV), myelodysplatic syndrome (MDS). *FLT3* gene mutant allele ratio indicated in parentheses. Variant allele frequency (VAF) indicated in parenthesis. * (asterisk) = translation termination (stop) codon.

## Data Availability

Data available upon request due to restrictions, privacy, and ethics.
